# Topics in Paediatrics. Haematology and Oncology

**Published:** 1981-07

**Authors:** D. Pearson


### *Book Reviews*

**Topics in Paediatrics. Haematology and Oncology.** Ed. P. H. Morris Jones (1980) London: Pitman Medical. 156 pp. £9.50 net.

These papers were presented at the Paediatric Conference at the Royal College of Physicians in October 1978. They are all an excellent contribution to the present thinking on the overall management of children with malignant disease. Each paper gives an up-to-date analysis of different aspects of childhood malignancy, and in the different tumour groups, the present aims of management. Important contributions in supportive care, both physical and psychological, are very welcome in a British meeting on this subject. All the authors are to be congratulated on the contribution this book can make to children with cancer. For those working in the field the most poignant and important paper was that by Mrs J. Davies on the Parent's Reaction. It must help many of us to improve our care of the parents as well as the child with malignant disease.

D. Pearson


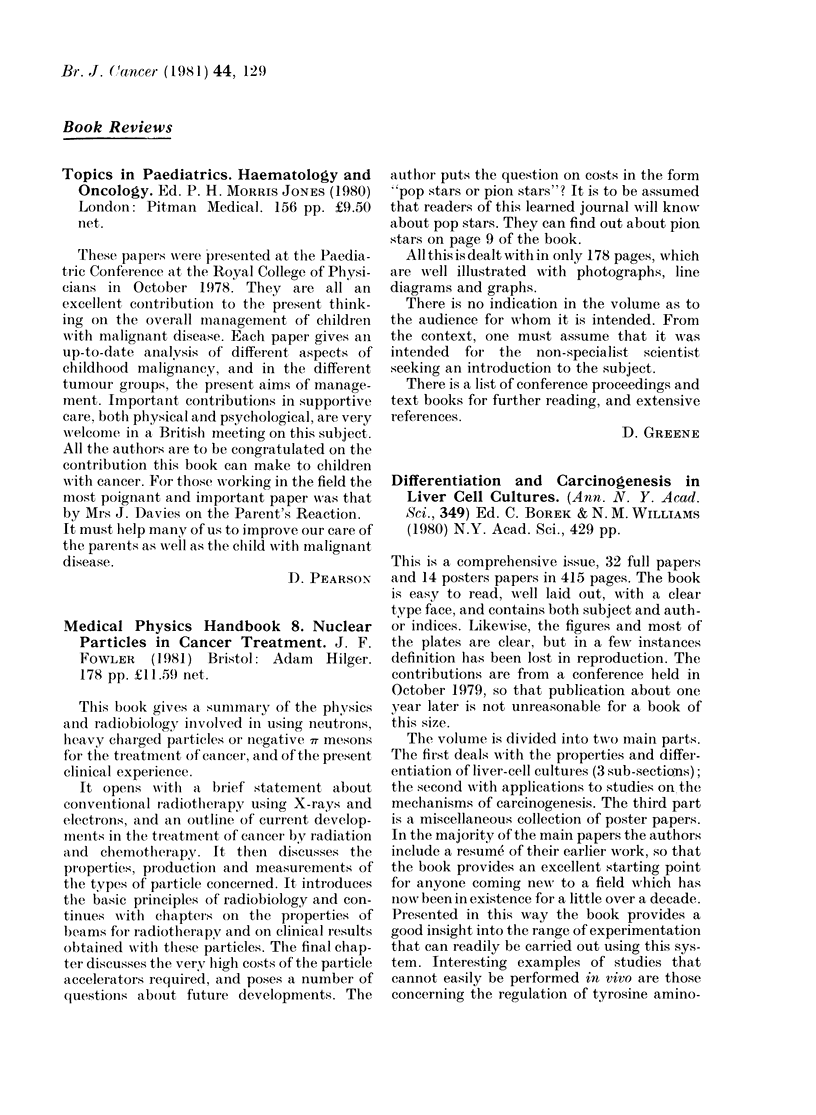


## References

[OCR_00004] Straatsma B. R. (1981). Jules Stein, M.D. 1896-1981.. Am J Ophthalmol.

